# Identification of peculiar gene expression profile in peripheral blood mononuclear cells (PBMC) of celiac patients on gluten free diet

**DOI:** 10.1371/journal.pone.0197915

**Published:** 2018-05-24

**Authors:** Moris Sangineto, Giusi Graziano, Simona D’Amore, Roberto Salvia, Giuseppe Palasciano, Carlo Sabbà, Michele Vacca, Marica Cariello

**Affiliations:** 1 Department of Interdisciplinary Medicine, “Aldo Moro” University of Bari, Bari, Italy; 2 National Cancer Research Center, IRCCS Oncologico Giovanni Paolo II, Bari, Italy; 3 Department of Biomedical Sciences and Human Oncology, Clinica Medica “A. Murri”, “Aldo Moro” University of Bari, Bari, Italy; 4 INBB, National Institute for Biostructures and Biosystems, Rome, Italy; Universite Clermont Auvergne, FRANCE

## Abstract

Celiac disease (CD) is a systemic disorder characterized by an immune-mediated reaction to gluten and a wide spectrum of clinical manifestations. Currently, the main treatment of CD is represented by adherence to a gluten-free diet (GFD) which determines the resolution of symptoms, and the normalization of the serology and of the duodenal villous atrophy. In the present study, we aimed to identify changes in gene expression in peripheral blood mononuclear cells (PBMCs) of celiac patients on GFD for at least 2 years, in order to identify novel disease biomarkers and candidate targets for putative therapeutic approaches. Microarray analysis was performed on PBMCs from 17 celiac patients on long-term GFD and 20 healthy controls. We identified 517 annotated genes that were significantly modulated between celiac patients and controls. Significant biological pathways were functionally clustered using the Core Function of Ingenuity System Pathway Analysis (IPA). Intriguingly, despite being on a GFD, celiac patients exhibited a peculiar PBMC profile characterized by an aberrant expression of genes involved in the regulation of immunity, inflammatory response, metabolism, and cell proliferation. Random forest algorithm was then used to validate the prediction ability of core genes as classifiers of the “celiac status”. In conclusion, our study identified a characteristic PBMCs signature profile in clinically asymptomatic celiac patient.

## Introduction

Celiac disease (CD) is an inflammatory, systemic disease characterized by an immune-mediated response triggered by dietary gluten exposition that leads to a variable damage of small-intestinal mucosa, a specific serum autoantibody response and a wide spectrum of clinical manifestations [1]. CD is one of the most common disease occurring in with 1% of the population worldwide, and predominantly affects infants and young children, although it may develop at any age in genetically susceptible individuals [2]. A combination of genetic predisposition, environmental factors (i.e. gluten diet) and cofactors (e.g. microbiota alterations, viral infections, drugs, wining age) determines the etiology of this disease [3]. The genetic predisposition is determined by HLA-DQ2 haplotype (DQA1*0501/DQB1*0201), which is expressed in 90% of celiac population, while 5% is represented by HLA-DQ8 haplotype (DQA1*0301/DQB1*0302) [1]. HLA class II genotype expressed on the cell surface of antigen-presenting cells plays an essential role in CD by presenting gliadin peptides to CD4+ Th1 cells, which trigger an immune response leading to the production of inflammatory cytokines, such as IFN-ɣ [4]. Nevertheless, despite CD is strongly associated with specific HLA class II genes, other factors may be important in the promoting disease onset also given that 30% of the general population expresses HLA DQ2; and other 39 celiac disease risk loci have been discovered [5]. During the pathogenesis of celiac disease an inflammatory cascade occurs, involving Th1 activation and an innate immune response in the intestinal mucosa, characterized by cytokines production such as interleukin 15 (IL-15), which promotes the differentiation of intraepithelial lymphocytes into cytotoxic CD8+ T cells [4]. A key role in lymphocyte proliferation/activation and enterocyte alteration is also played by nuclear factor kappa-light-chain-enhancer of activated B cells (NF-kB) pathways activation [6]. As a result of this pro-inflammatory cascade is the damage thus leading to villus atrophy and crypt hyperplasia. The main treatment for CD is the gluten free diet (GFD). Adherence to GFD for 6–26 months is able to promote the resolution of symptoms, the normalization of serology markers and duodenal villous atrophy [7]. Nevertheless, despite treatment, patients with CD are at higher risk of death for cardiovascular disease and cancer, as shown by Ludvigsson et al. in a wide retrospective Swedish cohort [8]. CD patients following GFD recommendation exhibit T lymphocytes specific to deamidated gluten peptides in the peripheral blood, which are able to produce pro-inflammatory cytokines such as IFN-gamma and IL-6 [9]. Therefore, a low grade undetectable pro-inflammatory status might be still present in GD patients despite GFD and might pathophysiologically explain the epidemiological association with CVR. In the present study we compared the whole genome expression profile of peripheral blood mononuclear cells (PBMCs) of celiac patients on GFD for at least 2 years to those of healthy subjects, in order to identify changes in the transcriptome that characterizes the celiac patients on GFD. PBMCs, the circulating immune cells mainly constituted by T lymphocytes (≤70%), B lymphocytes (≤15%), natural killer cells (≤10%), monocytes (≤5%), and dendritic cells (≤1%), [10]. play a key role in the inflammatory system, and the changes in their gene expression are considered predictors of the whole body inflammatory status in different conditions [10]. In the present study, we identified different gene expression profiles in PBMCs which could represent hallmarks of CD independent from GFD.

## Materials and methods

### Study population

Patients recruitment and clinical and biochemical assessment were collected at the Clinica Medica “A. Murri” (University Hospital of Bari, Italy). Seventeen celiac patients and twenty healthy subjects were recruited. The diagnosis of celiac disease was carried out at least two years before the study through measurement of antibodies and biopsy of duodenum, whose histology was evaluated by a pathologist and expressed according to Marsh-Oberhuber classification (Table 1). After 12 months of GFD, antibodies measurement and duodenal biopsy were performed, showing antibody negativity and normalization of the duodenal mucosa. During the study recruitment the patients were on GFD since at least two years (Table 1) and were newly submitted to antibodies assessment in order to exclude any positivity. The controls underwent the same biochemical analysis and they have never been biopsied. Baseline characteristics of subjects are described in Table 2. The presence of viral hepatitis, HIV infection, and daily alcohol consumption over 25 g/day were part of the exclusion criteria. In all subjects the past medical history and anthropometric parameters (weight, height and body mass index) were collected. All subjects underwent the physical examination. Blood samples were used for biochemical examinations, including the measurement of anti-transglutaminase antibodies (t-TG IgA and t-TG IgG), and anti-endomysium antibodies (EMA). Other peripheral blood samples were used for HLA II haplotype study and for PBMCs isolation. The study protocol was approved by the Ethical Committee of the Azienda Ospedaliero-Universitaria Policlinico di Bari, Italy. All patients gave their written informed consent for the use of clinical data and blood samples for scientific research purposes connected to this study.

**Table 1:** Characteristics of celiac patients.

Age (years)	35.3 ± 11.3
Gender	29.4% M (*n*5); 70.6% F (*n*12)
Time on GFD (years)	5.8 ± 3.8
HLA II haplotype	70.6% DQ2 (*n*12); 29.4% DQ8 (*n*5)
Marsh-Oberhuber grading	23.5% type 2 (*n4*); 11.8% type 3a (*n*2); 23.5% type 3b (*n*4); 41.2% type 3c (*n*7)
t-TG IgA (AU/mL)	100 ± 67.4
t-TG IgG (AU/mL)	12.5 ± 25
EMA	82.4% positive (*n*14); 17.6% negative (*n*3)

GFD: gluten free diet; *HLA*: Human leukocyte antigen; t-TG IgA: anti-transglutaminase IgA antibodies (cutoff: 10 AU/mL); t-TG IgG: anti-transglutaminase IgG antibodies (cutoff: 10 AU/mL); EMA: anti-endomysium antibodies.

**Table 2:** Clinical characterization of the study population.

Clinical variable	Control	Celiac	p-value
n	20	17	-
Weight (Kg)	68.03±10.45	59.97±10.27	NS
BMI (Kg/m^2^)	23.27±2.11	21.34±4.18	NS
Height (m)	1.71±0.08	1.66±0.06	NS
Glucose (mg/dl)	87.60±7.36	87.35±11.73	NS
Total cholesterol (mg/dl)	180.05±29.07	162.94±33.62	<0.05
HDL-c (mg/dl)	58.65±11.58	52.53±13.60	NS
LDL-c (mg/dl)	106.45±26.72	94.18±24.06	NS
TG (mg/dl)	69.70±32.47	71.94±36.35	NS
AST (U/I)	22.40±14.04	25.88±16.21	NS
ALT (U/I)	35.65±24.86	49.00±32.57	<0.05
GGT (U/I)	26.60±20.18	30.41±28.49	NS
ALP (U/I)	65.20±23.90	86.29±32.73	<0.05
Total bilirubin (mg/dl)	0.52±0.19	0.62±0.56	NS
Direct bilirubin (mg/dl)	0.13±0.08	0.13±0.07	NS
Indirect bilirubin (mg/dl)	0.39±0.17	0.49±0.49	NS
Creatinine (mg/dl)	0.81±0.16	0.73±0.18	NS
BUN (mg/dl)	3.56±0.67	4.17±1.03	NS
Uric acid (mg/dl)	33.65±7.65	29.00±6.23	NS
Total protein (g/dl)	7.27±0.39	7.39±0.60	NS
Albumin (g/dl)	4.22±0.26	4.08±0.27	NS
Sodium (mEq/l)	138.20±2.04	139.65±2.87	NS
Potassium (mEq/l)	3.93±0.35	4.19±0.34	<0.05
Magnesium (mg/dl)	1.84±0.20	1.99±0.15	<0.05
Calcium (mg/dl)	8.87±0.41	9.01±0.43	NS
ESR (mm/h)	5.60±4.39	8.29±5.60	NS
CRP (mg/l)	0.20±0.00	0.24±0.18	NS
Iron (ug/dl)	94.82±31.21	78.41±32.00	NS
Transferrin (mg/dl)	239.91±50.70	262.41±59.35	NS
Ferritin (ng/ml)	96.11±79.86	94.83±153.14	NS
PT INR	1.05±0.09	1.02±0.05	NS
LDH (U/L)	131.05±29.46	162.41±53.02	<0.01
TSH (mUI/L)	1.86±0.53	1.93±0.94	NS
FT3 (pg/ml)	2.99±0.23	3.05±0.45	NS
FT4 (ng/dl)	1.07±0.13	1.08±0.18	NS
Thyroglobulin (ng/ml)	13.73±9.88	7.16±4.73	<0.05
Ab anti TG (UI/ml)	3.10±6.56	46.77±72.55	NS
Ab anti TPO (UI/ml)	2.13±1.32	51.55±101.18	<0.01
HLA-DQ8	negative	positive (30%)	-
HLA-DQ2	negative	Positive (70%)	-
25-OH-D (ng/ml)	32.04±4.52	28.76±12.31	NS
Hemoglobin (g/dl)	13.87±1.15	12.91±1.54	NS
MCV (fl)	83.48±2.60	83.41±7.09	NS
WBC (10^3^/μl)	6.28±1.32	5.97±1.52	NS
Monocytes (%)	6.39±1.55	6.54±2.11	NS
Lymphocytes (%)	35.25±8.71	36.41±6.64	NS
Neutrophils (%)	54.31±8.98	54.15±7.07	NS
Basophils (%)	0.51±0.24	0.41±0.18	NS
Eosinophils (%)	3.44±1.54	2.35±1.72	<0.01

BMI: body mass index; HDL-C: high density lipoprotein cholesterol; LDL-C: low density lipoprotein cholesterol; TG: triglycerides; AST: aspartate aminotransferase; ALT: alanine aminotransferase; GGT: ɣ-glutamyltranspeptidase; ALP: alkaline phosphatase; BUN: blood urea nitrogen; ESR: erythrocyte sedimentation rate; CRP: C-reactive protein; PT-INR pro-thrombin international normalised ratio; LDH: lactate dehydrogenase; TSH: thyroid stimulating hormone; FT3: free triiodothyronine; FT4: free thyroxine; Ab anti TG: anti thyroglobulin antibodies; Ab anti TPO: anti thyroperoxidase antibodies; MCV: mean corpuscular volume; WBC: white blood cells.

### Biochemical measurements

After overnight fasting, serum was collected for the evaluation of standard biochemical markers of glucose and lipid metabolism, liver and renal function, complete blood count, iron status and inflammation by standard biochemical methods using a standard, previously validated protocol [11]. Moreover, t-TG IgA and t-TG IgG were measured by enzyme-linked immunosorbent assay (automated analyser, Dynex Technologies, Chantilly, VA USA), while EMA positivity was assessed by indirect immunofluorescence.

### Sample collection and PBMC isolation

Fresh whole blood (18 ml) was collected by standardized venepuncture in EDTA anti-coagulant tubes (Vacuette®, Greiner Bio-One, Kremsmunster, Austria). PBMCs were isolated at once, after collection of blood samples, using standard and previously validated protocol [11;12]. PBMC were isolated by density separation over a Ficoll-Paque (GE healthcare, Sweden) gradient.

### RNA extraction and reverse-transcription

Total RNA was extracted from the PBMCs pellet by QIAzol® Lysis Reagent (Qiagen, Hilden, Germany), according to the manufacturer’s instructions using standard and previously validated protocol [11]. To avoid possible DNA contamination, the RNA was treated with DNAase-1 (Ambion, Foster City, CA). RNA purity (A260/A280 > 1.75), and the concentration was checked by spectrophotometer, while the RNA integrity was assessed by Bio-RAd ExperionTM (Bio-Rad, Hercules, CA). Only the samples with Relative Quality Index (RQI) > 8 were used for reverse-transcription. According to the manufacturer’s instructions, cDNA was synthesized by reverse-transcribing 4 μg of total RNA in a volume of 100 μl using the High Capacity DNA Archive Kit (Applied Biosystems, Foster Cyti, CA). For TaqMan gene validations, due to the small quantity of RNA achieved, we used a High Capacity RNA-to-cDNA Kit (Applied Biosystems, Foster City, CA) to reverse-transcribe 10 ng of total RNA in a volume of 20 μl.

### Microarray analysis for gene expression profiling

We conducted microarray gene expression analysis on total RNA (400 ng) extracted from PBMCs. amplified using the Illumina Total Prep RNA Amplification kit (Ambion, Austin, TX, US) following the manufacturer’s instructions. For the whole-genome expression we used the Illumina whole genome direct hybridization assay (Illumina HumanHT-12-V3’ Expression Kit) on the Illumina microarray platform (Illumina iScan System) using standard and previously validated protocol [13]. Data were processed using the Illumina Genome Studio Software through specific algorithms of filtration and cleaning of the signal [13]. Data were normalized with the quantile method. Final output consisted of normalized fluorescence intensity of each probe (AVG signal), representing the expression levels of each gene. Genes with AVG signal lower/equal to the background or with detection p value.0.001 was excluded. We also excluded genes discontinued or poorly annotated according to NCBI Entrez Gene Database records. We thus performed pathways analysis on a final number of 517 significant genes (Fold.1.5; p<0.05) using the ‘‘Core Analysis” function of Ingenuity Pathway Analysis (Ingenuity System Inc., USA) to identify networks and pathways modulated in GFD celiac patients vs healthy controls. Microarray data are available on GEO (Ref: GSE113469; https://www.ncbi.nlm.nih.gov/geo/query/acc.cgi?acc=GSE113469).

### Quantitative real-time polymerase chain reaction (RTqPCR)

PCR assays were performed in 96 well optical reaction plates using the ABI 7500HT system (Applied Biosystems, Foster City, CA) using standard and previously validated protocol [11]. PCR assays were conducted in triplicates for each sample. To validate our microarrays results, we used TaqMan® Gene Expression Assays and TaqMan® Universal PCR Master Mix with UNG following the manufacturer’s instructions. The following PCR conditions were used: UNG incubation at 50°C for 2 min (only for TaqMan); then (for all the experiments) denaturation at 95°C for 10 min, followed by 40 cycles at 95°C for 15 s, and then at 60°C for 60 s. Baseline values of amplification plots were set automatically, and threshold values were kept constant to obtain normalized cycle times and linear regression data. Relative quantification was performed using the ΔΔCT method. Values were plotted as fold change ± SEM.

### Statistical analysis

In order to evaluate differences between the two groups of interest the normalized data exported from genome studio software were analyzed with Mann-Whitney *U* test while a correlation between continuous variables were assessed using the Pearson’s correlation coefficient. p-values < 0.05 were considered statistically significant. A fold induction of ±1.5 was considered the minimum acceptable cut-off and the raw p-values were adjusted by the Benjamini-Hochberg procedure to control the False Discovery Rate (FDR) [14;15]. We considered “biologically relevant” (15) only genes statistically significant and highlighted by pathway analysis. To study the ability of this set of genes to characterize celiac patients from controls and to mitigate the impact of small sample size on the study conclusions the more robust and innovative Random Forest Analysis was applied. A Random Forest (RF) is a classification algorithm consisting of an ensemble technique that overcomes the problem of a low number of observations [16;17]. The principal characteristics of RF are the identification and classification of relevant differentially expressed genes [16] and the estimation of the error rate related to their predictive ability. This efficient approach, considering that a RF with a low error rate (high C-Index) is a strong classifier, gave us the possibility to define an “identity card” of the genes characterizing celiac patients.

The ROC curve analysis was applied to the selected genes (5 up-regulated, 5 down-regulated) in order to estimate their ability to discriminate celiac patients from healthy controls. The area under the ROC curve (AUC) was used as a measure of the capability of each gene to distinguish between the two clinical groups.

All the analyses were performed using the SAS Package (Release 9.1) and the R Package (Version 2.12.2).

## Results

### Clinical characterization of the study population

Seventeen celiac patients on GFD for at least 2 years were compared to twenty healthy controls. We show the baseline characteristics of the study population in Table 2. Susceptibility to celiac disease is linked to the presence of HLA-DQ2 and HLA-DQ8 heterodimers. Accordingly, 70% of the celiac patients in our population presented HLA-DQ2 heterodimer and 30% HLA-DQ8 heterodimer, while these genetic markers were completely absent in healthy subjects. Celiac disease is a systemic disease characterized by a specific serum autoantibody profile and a wide spectrum of clinical manifestations. Celiac population on GFD exhibited a normal serum autoantibody profile (Table 1). Although within normal values, alkaline phosphatase (ALP), alanine aminotransferase (ALT), potassium, magnesium, lactate dehydrogenase (LDH) and anti thyroperoxidase antibodies (Ab anti-TPO) levels resulted increased while total cholesterol, thyroglobulin and eosinophils were significantly lower in celiac patients compared to controls.

### Whole genome analysis in PBMCs of celiac patients on GFD

**Fig 1:**
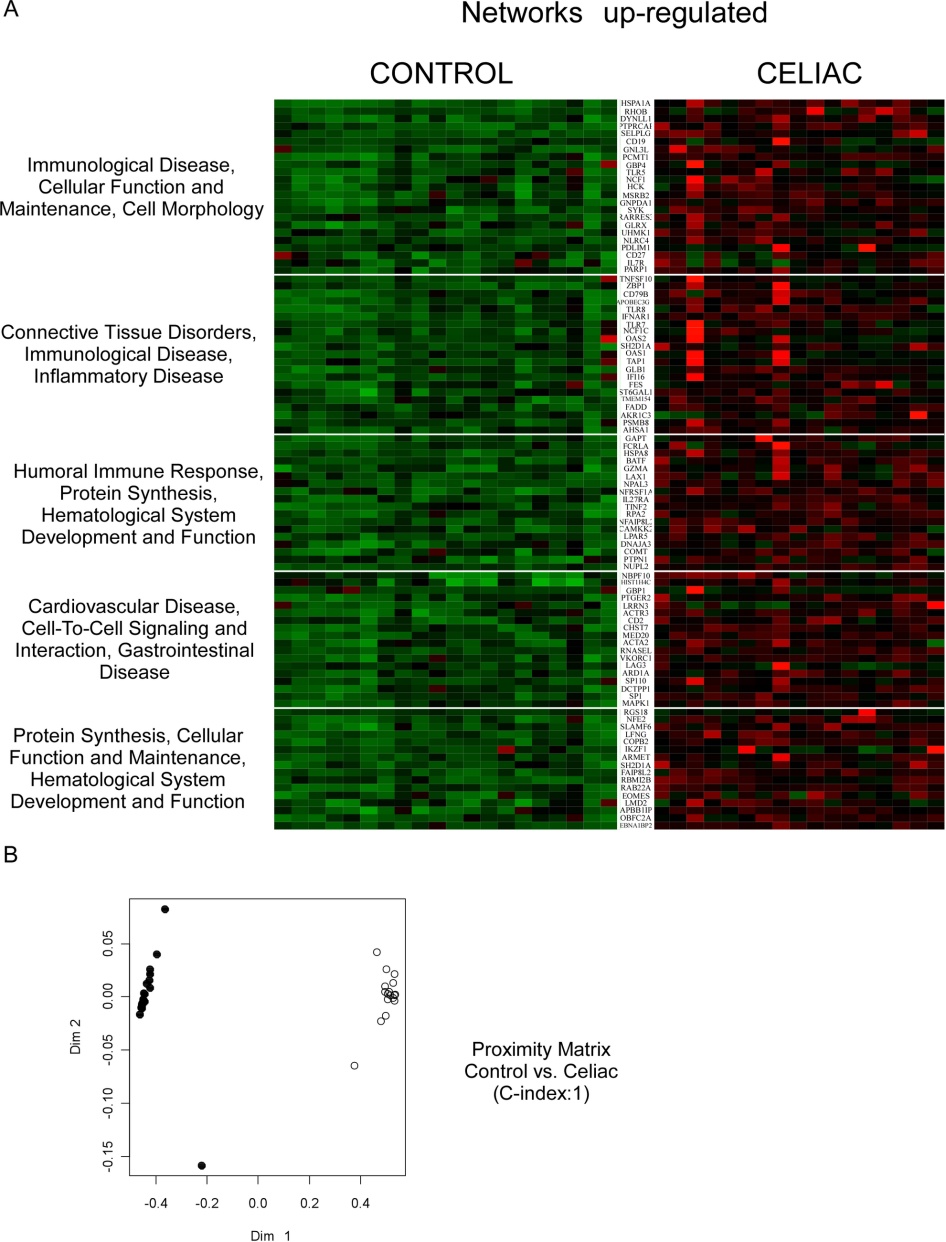
Gene expression profiling in GFD celiac patients. (A) Genes up-regulated in GFD celiac patients were profiled using microarrays, and clustered in large networks displaying a coordinate biological function. Data are shown in a heatmap with a matrix format of the genes differentially modulated within the specific network; single rows represent gene expression in a single patient (column). Colours: red, expression greater than the mean; black, expression equal to the mean; green, expression smaller than the mean. Lateral bars: fold changes among groups. (B) Proximity matrix of the RF algorithm: RF discriminated patients with celiac disease on GFD from control patients in 100% of cases (C-Index = 1). Legend: (black) controls; (white) celiac patients.

**Fig 2:**
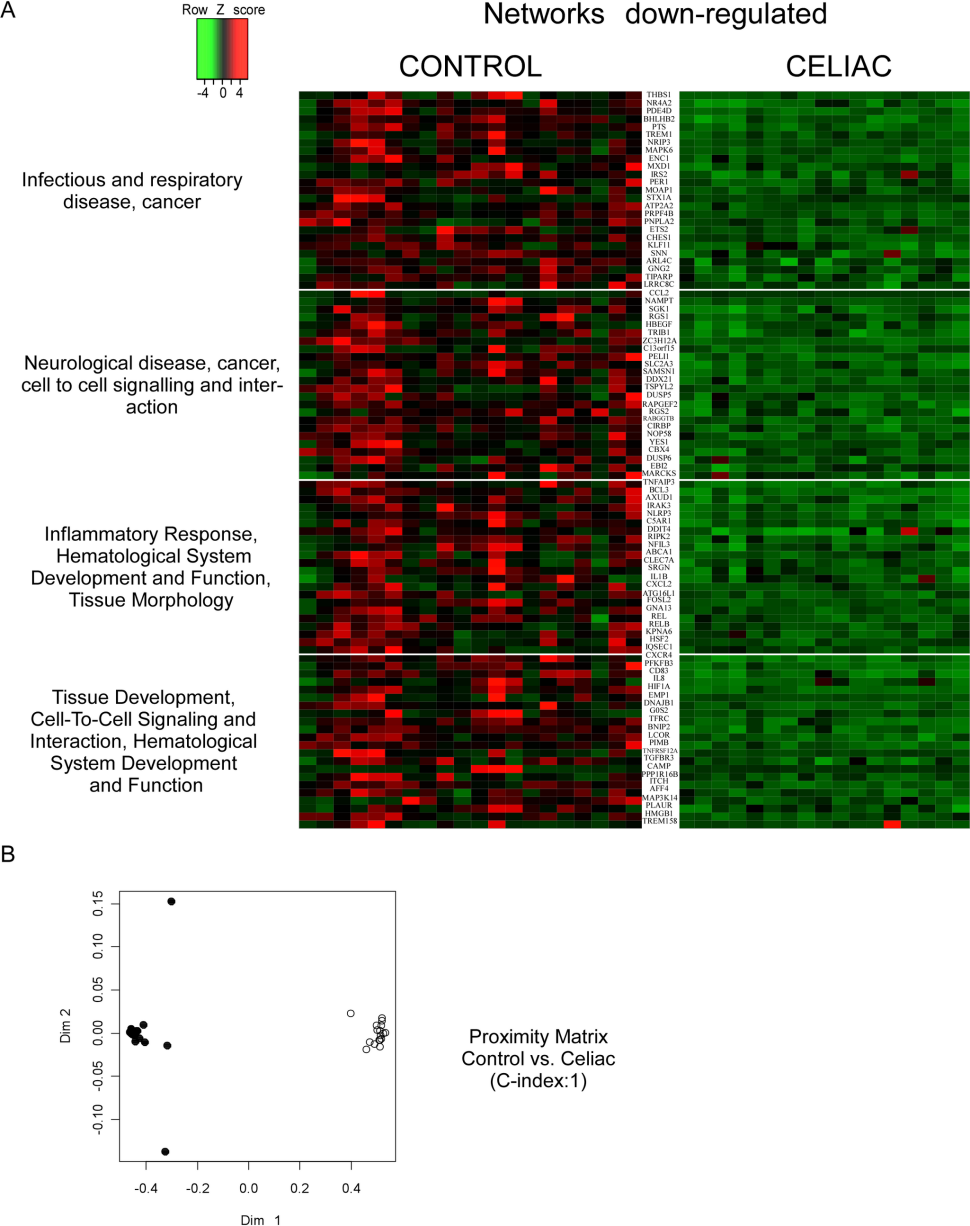
Gene expression profiling in GFD celiac patients. (A) Genes down-regulated in GFD celiac patients were profiled using microarrays, and clustered in large networks displaying a coordinate biological function. Data are shown in a heatmap with a matrix format of the genes differentially modulated within the specific network; single rows represent gene expression in a single patient (column). Colours: red, expression greater than the mean; black, expression equal to the mean; green, expression smaller than the mean. Lateral bars: fold changes among groups. (B) Proximity matrix of the RF algorithm: RF discriminated patients with celiac disease on GFD from control patients in 100% of cases (C-Index = 1). Legend: (black) controls; (white) celiac patients.

We used whole-genome microarray analysis to analyze global changes to the transcriptome in PBMCs from celiac patients on GFD. Overall, the signal intensity of 4482 genes resulted statistically different by the Mann-Whitney *U* test in celiac (vs. control), thus underscoring a profound diversity in the activation status of the PBMCs of celiac patients on GFD when compared to healthy controls. In order to evaluate the peculiarities of celiac patients vs controls, we selected 517 annotated genes (261 up-regulated and 256 down-regulated) that were significantly different in celiac population compared to healthy subjects (P<0.01; FDR<0.01). To provide an interpretation of our results, significant biological pathways were functionally clustered using the Core Function of Ingenuity System Pathway Analysis (IPA). We considered ‘biologically relevant’ only those ‘statistically significant’ genes included in ‘significantly modulated’ pathways and networks (Figs 1A and 2A). The profiling of CD patients revealed a “pro-inflammatory” shift as shown confirmed by the dysregulation of pathways involved in the control of the immune system and inflammatory response. The Random Forest (RF) algorithm was used to validate the prediction ability of “celiac status” of the selected gene set The accuracy of core genes in classifying CD patients was estimated by the algorithm as high as 100% (C-Index 1; Figs 1B and 2B) thus suggesting that CD PBMCs have a specific transcriptome signature when compared to healthy controls.

### “Identity card” of celiac patients on gluten free diet for at least 2 years

**Fig 3:**
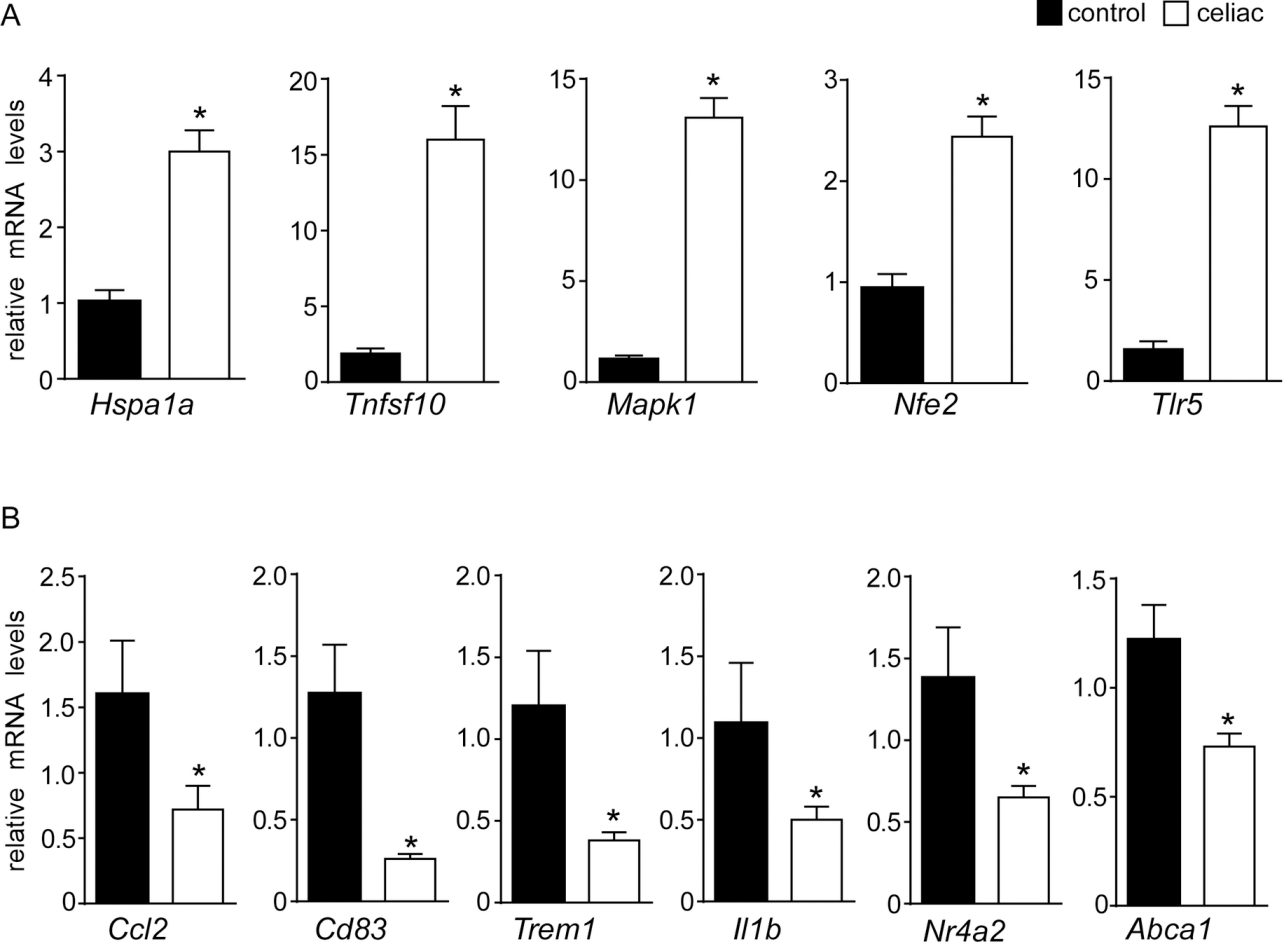
**RT–qPCR confirmations**: relative mRNA expression of (A) HSPA1a, TNFSF10, MAPK1, NFE2, TLR5; (B) ABCA1, CCL2, CD83, TREM1, IL1β and NR4A2. Cyclophilin was used as reference gene, and values were normalized to data obtained from control patients. Data were plotted as fold change ± SEM (* = p ≤ 0.05).

In order to evaluate the biological meaning of the changes in GE of PBMCs in CD, we analyzed the genes differentially expressed among the groups using IPA pathways, network and “upstream regulator” prediction tools (Figs 1A and 2A). By using a validated quantitative real-time PCR method, we assessed the mRNA expression of 11 genes (5 up-regulated and 6 downregulated) involved in the modulation of immunity system, cell proliferation and metabolism. We observed a significant increase in the mRNA levels of mitogen-activated protein kinase 1 (MAPK1), heat shock protein 70 (HSPA1a), toll-like receptor 5 (TLR-5) and tumor necrosis factor superfamily member 10 (TNFSF10) involved in both innate and adaptive immune responses (Fig 3A). Moreover, we also confirmed the up-regulation of nuclear factor erythroid-derived 2 (NFE-2), an essential factor in the maturation of platelets and red cells (Fig 3A). In line with these results, we validated the down-regulation of chemokine (C-C Motif) ligand 2 (CCL2), CD83, triggering receptor expressed on myeloid cells (TREM1) and interleukin 1b (IL-1b) in celiac patients compared to control group (Fig 3B). The involvement of these molecular patterns points to an intriguing activation of innate immune pathways in celiac patients despite long-term GFD. Finally, we observed a downregulation of ATP-binding cassette A1 (ABCA1), a gene that promotes cholesterol efflux leading to HDL formation, and NR4A2 (NURR1), a nuclear receptor involved in inflammation in microglia and astrocytes (Fig 3B).

**Fig 4:**
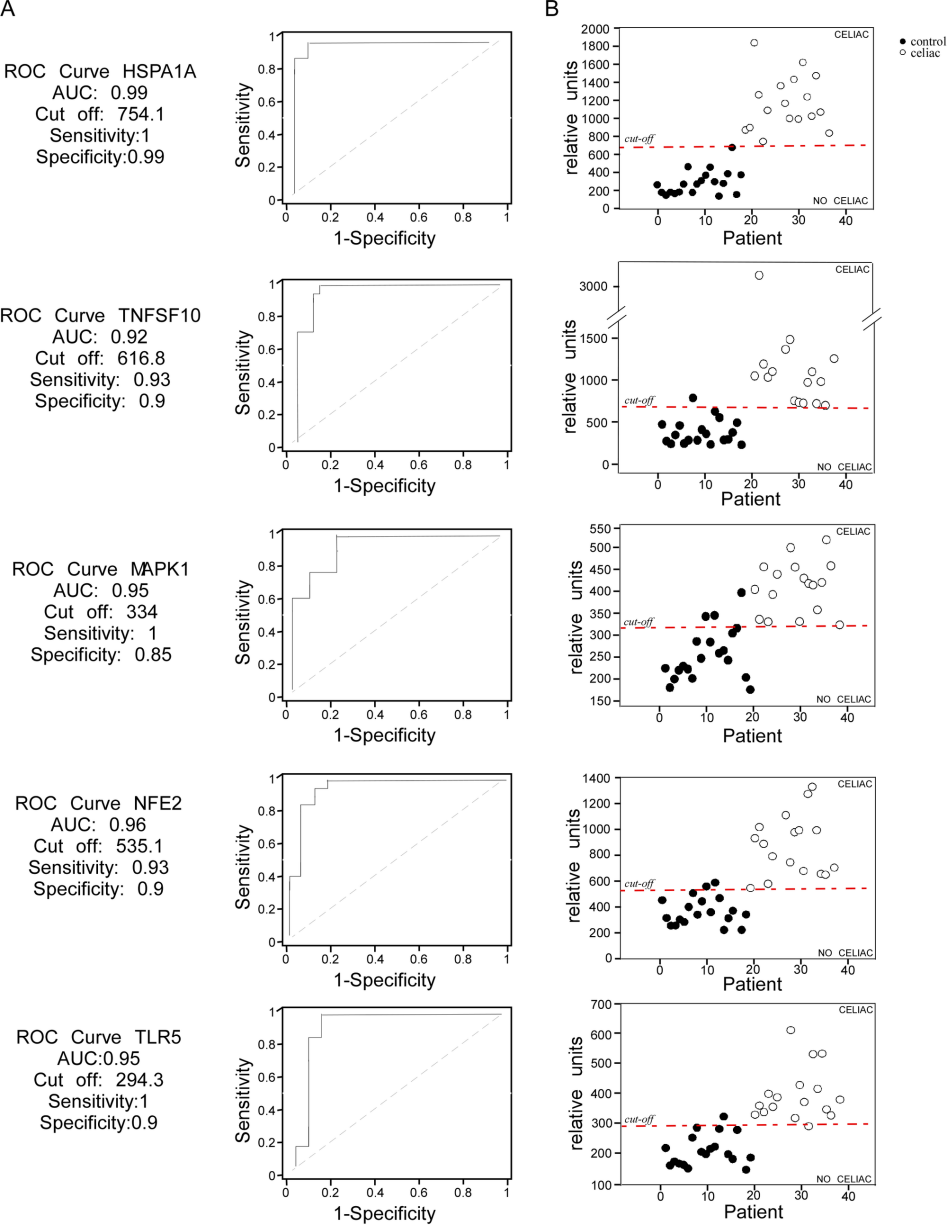
Up-regulated genes in PBMCs as candidate biomarkers of celiac patients on GFD. (A) The ROC curves were characterized by specificity and sensitivity. (B) The cut-off values obtained with the ROC curves were able to discriminate GFD celiac patients from controls. Each patient is represented by a dot. Legend: (black) controls; (white) celiac patients.

**Fig 5:**
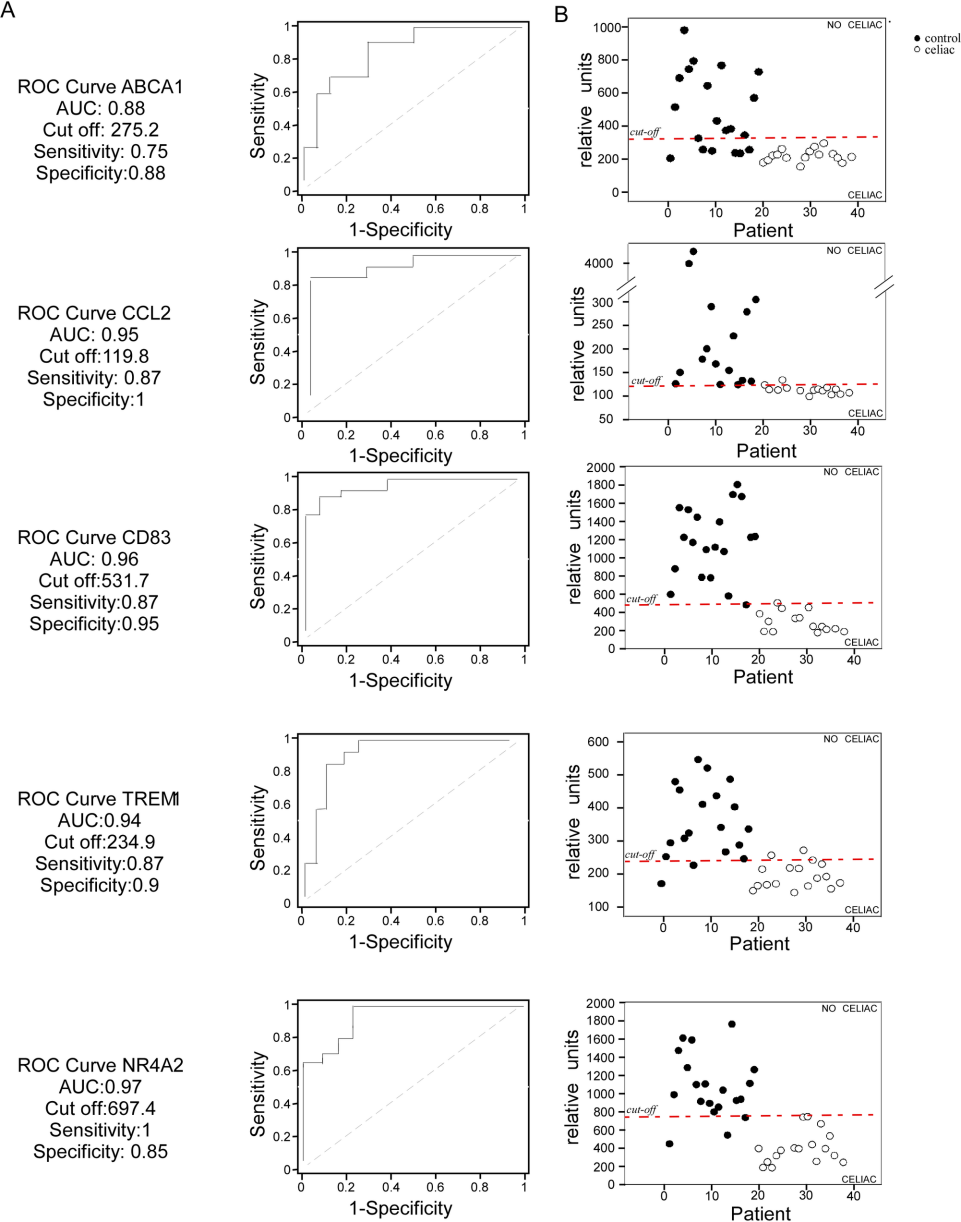
Down-regulated genes in PBMCs as candidate biomarkers of celiac patients on GFD. (A) The ROC curves were characterized by specificity and sensitivity. (B) The cut-off values obtained with the ROC curves were able to discriminate GFD celiac patients from controls. Each patient is represented by a dot. Legend: (black) controls; (white) celiac patients.

In order to prove the ability of the up-regulated and down-regulated gene sets to discriminate the “celiac status”, we calculated the ROC curves (Figs 4A and 5A). Up- and down-regulated genes were characterized by high specificity and sensibility in predicting the “celiac status”. Particularly, HSPA1a and CCL2 cut-off values were able to classify celiac status with high accuracy (Figs 4B and 5B) confirming a specific gene expression “identity card” of GFD celiac patients.

## Discussion

Celiac disease is a gluten-sensitive autoimmune disease affecting genetically susceptible individuals, which results in small-intestinal mucosal injury leading to malabsorption and malnutrition-related problems, including chronic diarrhea, bloating, weight loss, anemia, iron deficiency, headache, chronic fatigue, and osteoporosis [18]. Genetic predisposition (HLA-DQ2 and HLA-DQ8 haplotypes), with positive duodenal biopsy and serological antibodies upon gluten contained diet are required for the diagnosis of CD [18;19]. In most cases the gluten free diet alone is able to restore villous atrophy and normalize serology markers in celiac patients, who are thus considered “healthy subjects”, although some authors reported that the mortality risk among patients with CD or latent CD is modestly increased [8;20].

In the present work, we compared the PBMCs transcriptome of celiac patients on long term GFD, characterized by negative antibodies and normal duodenal mucosa, with healthy subjects in order to identify a specific gene expression pattern of CD. The clinical parameters of the population were analyzed and, as expected, we found significant differences between celiac group and controls. In particular, we observed raised anti thyroperoxidase and thyroglobulin antibodies (Table 2) in the celiac group thus confirming the well-known association between CD and autoimmune thyroiditis [21]. Interestingly, we profiled 517 differentially expressed genes (261 up-regulated and 256 down-regulated) in the celiac population involved in the modulation of the immune system, the inflammatory response and metabolism, and the pathogenesis of cardiovascular disease and cancer (Figs 1A and 2A). These genes were able to discriminate the two populations with 100% of accuracy (Figs 1B and 2B), shedding light, for the first time, on a peculiar transcriptome signature in PBMCs of celiac patients. A recent study demonstrated that PBMCs of celiac patients had a systemic activation and high cytokine expression levels, especially IFN-γ, IL-4 and IL-8 while celiac patients on GFD for 1 or 2 years restored normal levels [22]. Therefore, we focused on biologically relevant genes and we observed an up-regulation of HSPA1a, MAPK1, TLR-5, TNFSF10 and down-regulation of CCL2, CD83 and IL-1β. HSPA1a gene encodes for heat shock protein (HSP) 70–1 located in the MHC class III region. This gene is particularly interesting since the HSP70-1 seems to be overexpressed in CACO-2 cells after gliadin exposure, mediating the cytoskeletal and tight junction repair during recovery stage in intestinal cells [23]. It is also involved in autoimmune-like systemic disorder and cancer [24]. Moreover, HSP-70 overexpression in mouse fibrosarcoma cells was demonstrated to confer resistance to killing by immune cells [25] and it was correlated with Ki-67 positivity in lung cancer [26] as well as with increased tumor grade and poor prognosis [27;28]. HSP-70 inhibits the intrinsic and extrinsic apoptosis pathways blocking BAX translocation to mitochondria. A study performed on Navarra’s community suggested that HSP-70 was a predisposing gene to develop the CD clinical symptoms as the authors found an association between HSP-70 gene polymorphisms and celiac disease [29;30]., With regards to inflammation, we found a drammatic up-regulation of TLRs pathway genes, especially of TLR5, which is involved in bacterial flagellin recognition [31] and in the onset of many diseases such as inflammatory bowel disease. Furthermore, TLRs overexpression has been associated to celiac disease [32], and accordingly to this observation, the stimulation of PBMCs from celiac patients with pepsin digest of wheat gliadin fraction led to IL-1β secretion via TLR4/MyD88/TRIF/MAPK/NF-kB signaling pathway [33].

Conform to our hypothesis, we have found the upregulation of MAPK1 in GFD celiac subjects. An aberrant activation of the MAP kinase pathway has been demonstrated in PBMCs of CD patients with active disease [34], indeed MAP kinase cascade plays a regulatory role in both innate and adaptive immune responses [35;36]. Therefore, the significant differences in the transcriptome between the two study groups confirm the hypothesis that celiac subjects preserve the “genetic memory” of the active disease despite GFD. This memory is expressed by the aberrant transcription of genes involved in cellular repair and apoptosis regulation like HSPA1a, and an important alteration of innate immunity characterized by the hyper-activation of TLRs-MAPKs axis.

On the other hand, the downregulation of genes involved in innate acute immune response triggered by gliadin ingestion, such as CCL2, CD83 (marker of human dendritic cells), IL-1β and TREM1 was also observed. Raised levels of chemokines like monocyte chemoattractant protein-1 (MCP-1 also called CCL2) and pro-inflammatory mediators like IL-1β [37;38] have been observed in the primary immune response to gluten in CD patients [39;40]. TREM-1 is expressed mainly on the surface of granulocytes, monocytes and neutrophils [41;42] and its induction leads to activation of a cascade of intracellular events resulting in MCP-1 and IL-1β production. Our data suggested that whilst GFD is unable to counteract the activation of TLRs pathway can normalize cytokines production and the adaptive immunity recruitment as suggested by the down-regulation CCL2, IL-1β and CD83. Also, given the crucial role of TREM-1 in the regulation of innate immunity since it is a synergist of TLRs signaling cascade activated by LPS and other ligands [42;43]. Its down-regulation might be a compensative attempt to counteract the over-activation of TLRs seen in GFD celiac patients resulting in no cytokines and chemokines production. However, further studies are needed to clarify this point.

In summary, this study unveiled a specific “identity card” of GFD celiac patients, which differentiates them from healthy subjects. In fact, these patients exhibited a peculiar PBMC gene expression profile suggesting increased cellular repair and proliferation and dysfunctional innate immunity, which could make these subjects susceptible to autoimmune diseases and cancer.

In order to improve the therapy strategies, further studies are necessary to understand why celiac patients on gluten free diet exhibited a different gene expression profile in PBMCs compared to control subjects. A possible explanation is that GFD celiac patients were not “strictly” adherent to diet. In line with this, a recent study has shown that patients with celiac disease trying to follow a GFD have imperfect knowledge of the gluten content of common foods which they may encounter [44]. In this light, further research is warranted to better investigate the nutritional habits of celiac patients (e.g. trough specific questionnaires) as well as studies including control subjects on GFD to clarify the role of gluten on gene expression profile. Furthermore, in the present study controls were HLA DQ2/DQ8 negative; whilst this allowed to exclude any pro-inflammatory profile due to possible gluten reactivity, on the other hand represented a significant difference between the two study groups. It has been demonstrated that the intestinal epithelial cells from subjects with only family history of CD produce more HSP70 and IL-15 than controls, while intraepithelial cytotoxic T cells expressed higher levels of activating NK receptors than controls, although lower when compared to active CD [45]. The genetic background of celiac patients relatives was thus sufficient to have a different inflammatory pattern in the intestinal mucosa when compared to controls. In conclusion, in the present study, we depicted a comparative whole genome expression chart from PBMCs in GFD celiac patients and in healthy subjects. This strategy allowed us to identify a set of genes characterizing GFD celiac patients as well as novel putative biomarkers of celiac status.
